# Supplementary material for: Pathogenesis-adaptive polydopamine nanosystem for sequential therapy of ischemic stroke
Source: Nat Commun. 2023 Nov 6;14:7147. doi: 10.1038/s41467-023-43070-z (PMC10628287; doi:10.1038/s41467-023-43070-z)
Supplement: Supplementary file 1 — Supplementary Information [file 41467_2023_43070_MOESM1_ESM.pdf]

Supplementary Information

Pathogenesis-adaptive polydopamine nanosystem for sequential therapy of ischemic stroke

Di Wu[1†*], Jing Zhou[1†], Yanrong Zheng[1†], Yuyi Zheng[1], Qi Zhang[1], Zhuchen Zhou[1], Xiaojie Chen[1], Qi Chen[1], Yeping Ruan[1], Yi Wang[1,2], Zhong Chen[1*]

[1] Key Laboratory of Neuropharmacology and Translational Medicine of Zhejiang Province, School of Pharmaceutical Sciences, Zhejiang Chinese Medical University, Hangzhou 310053, China.

[2] Zhejiang Rehabilitation Medical Center Department, The Third Affiliated Hospital of Zhejiang Chinese Medical University, Hangzhou 310053, China.

[†] These authors contributed equally to this work.

[*] Corresponding author. Di Wu: wudichem@zju.edu.cn; Zhong Chen: chenzhong@zju.edu.cn

Abbreviation list

ALT---------alanine aminotransferase

AST---------aspartate aminotransferase

Bax---------Bcl-2-associated X protein

BBB---------blood-brain barrier

Bcl-2---------B-cell leukemia-2

BET---------Brunauer-Emmett-Teller

BrdU---------5-bromodeoxyuridinc

DCX---------doublecortin

DDSs---------drug delivery systems

EB---------Evans Blue

GFAP---------glial fibrillary acidic protein

HPLC---------high performance liquid chromatography

KEGG---------Kyoto Encyclopedia of Genes and Genomes

LRP-1---------lipoprotein receptor-related protein-1

MCAO---------middle cerebral artery occlusion

Mino---------minocycline

MMP---------matrix metalloproteinase

mPDA---------mesoporous polydopamine

mPDA-Pep-Mino---------unmodified drug-loaded mesoporous polydopamine

OGD---------oxygen and glucose deprivation

PEG---------polyethylene glycol

Pep---------MMP-2 responsive peptide. Sequence: Ac-CSSSGPLGIAGQSSS

ROS---------reactive oxygen species

T-mPDA---------RAP-12-modified mesoporous polydopamine

T-mPDA-Pep-Mino---------RAP-12-modified drug-loaded mesoporous polydopamine

TNF-α---------tumor necrosis factor-α

TTC---------2,3,5-triphenyltetrazolium hydrochloride

TUNEL---------transferase mediated nick end labeling

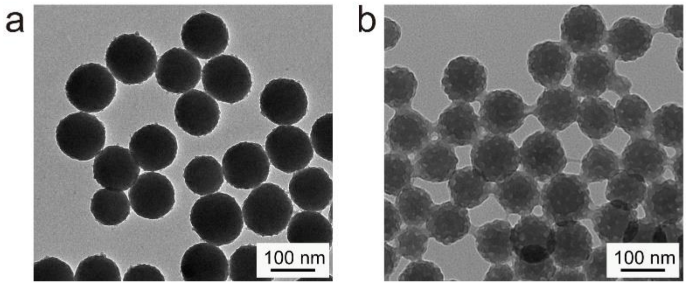

Supplementary Figure 1. TEM images of non-mesoporous and mesoporous polydopamine nanoparticles. More than 10 times are repeated independently with similar microscopy images.

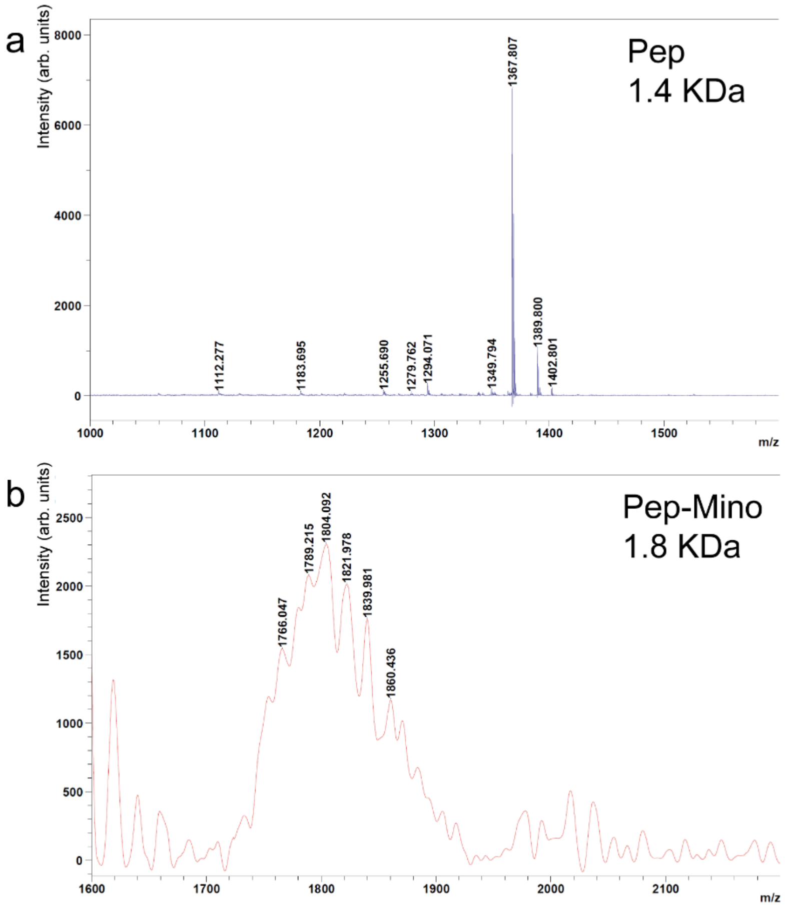

Supplementary Figure 2. Analysis of molecular weight of unconjugated Pep (1367.8 Da) and Pep-Mino (1804.1 Da) confirmed by MALDI-TOF-MS.

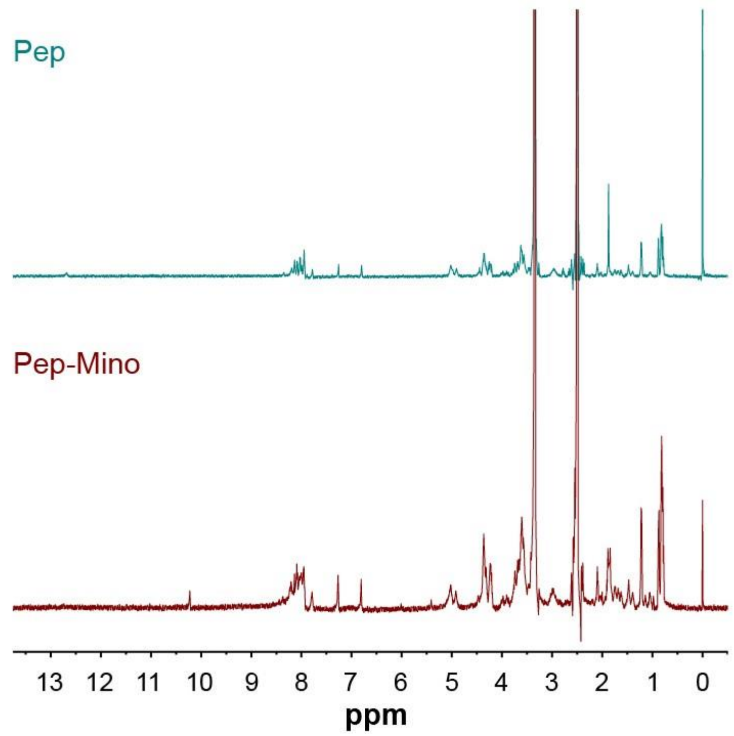

Supplementary Figure 3. ^{1}H-NMR spectrum of unconjugated Pep and Pep-Mino.

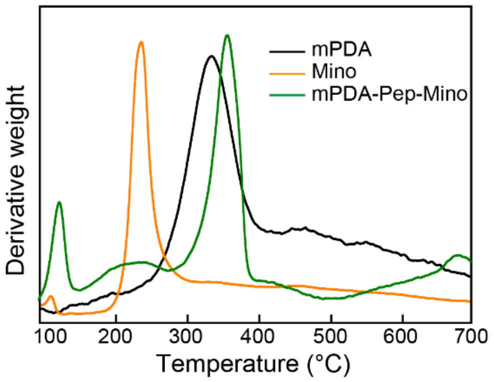

Supplementary Figure 4. Derivative thermogravimetry results of mPDA, Mino, and mPDA-Pep-Mino from 100 to 700 °C. Source data are provided as a Source Data file.

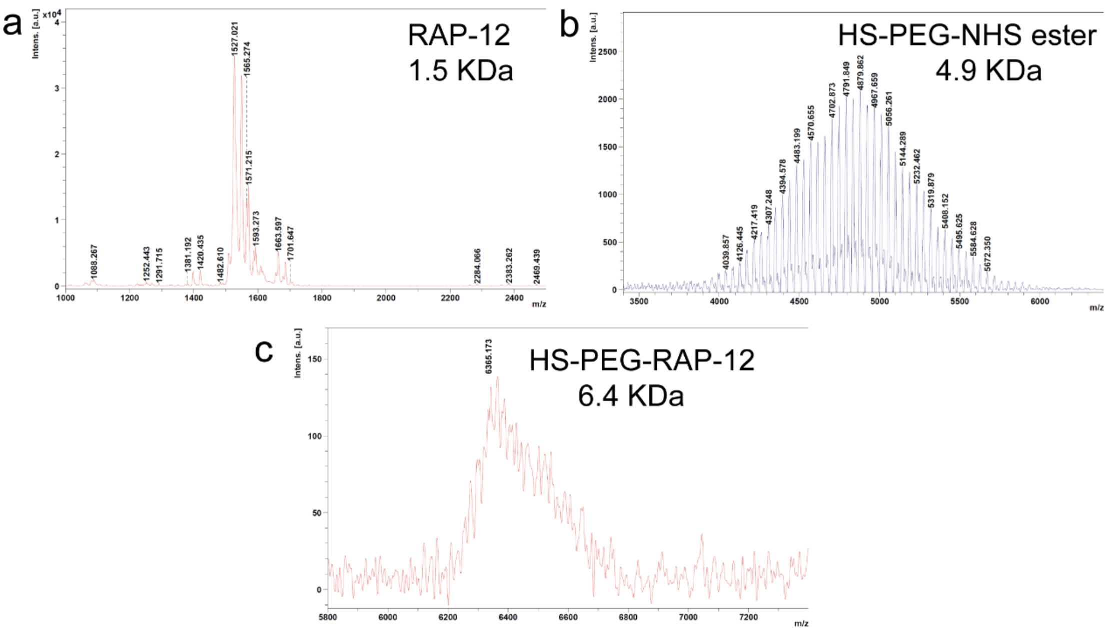

Supplementary Figure 5. Analysis of molecular weight of brain-targeted RAP-12 (1527.0 Da), thiol-PEG NHS ester (4879.9 Da), and PEGylated RAP-12 (6365.2 Da).

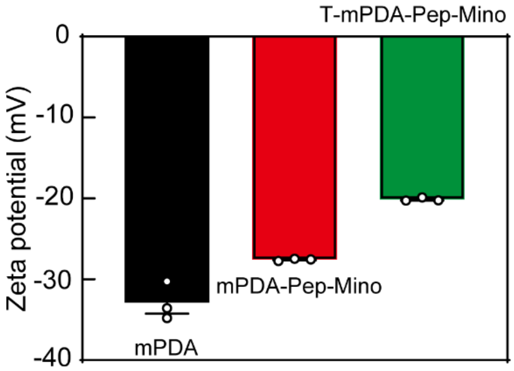

Supplementary Figure 6. Surface charge analysis of different nanoparticles including mPDA (-32.88 ± 2.34 mV), mPDA-Pep-Mino (-27.58 ± 0.14 mV), and T-mPDA-Pep-Mino (-20.12 ± 0.22 mV) ($n = 3$ independent samples). The data are presented as means ± SEM. Source data are provided as a Source Data file.

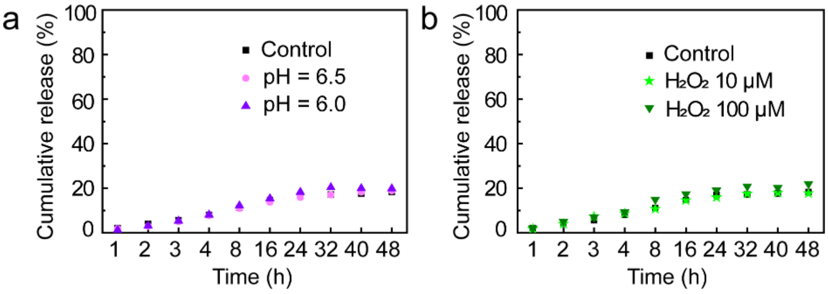

Supplementary Figure 7. Cumulative drug release of the nanosystem in acidic conditions (pH = 6.5 and pH = 6.0) or in the presence of H_2O_2 (10 and 100 μM).

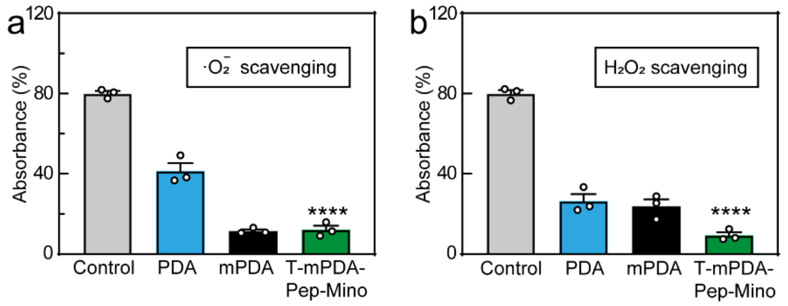

Supplementary Figure 8. Evaluation of **a** $\cdot O_2^-$ and **b** H_2O_2 scavenging by different nanoparticles at the same particle concentration (*n* = 3 independent samples). The data are presented as means ± SEM.Source data are provided as a Source Data file. (P****<0.0001 compared with the control group)

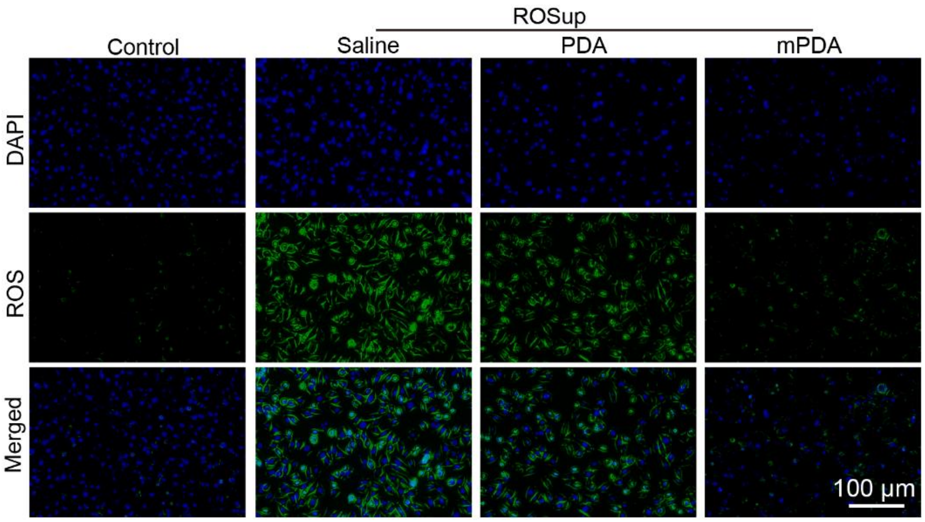

Supplementary Figure 9. In vitro ROS scavenging of PDA and mPDA nanoparticles in SH-SY5Y cells. ROS level was observed by using DCFH-DA probes and the cell nuclei were stained by DAPI.

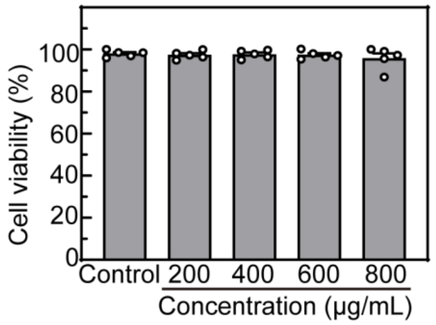

Supplementary Figure 10. Cell viability of SH-SY5Y cells incubated with the nanoparticles at different concentration up to 800 μg/mL confirmed by CCK-8 assay (*n* = 5 independent samples). The data are presented as means ± SEM. Source data are provided as a Source Data file.

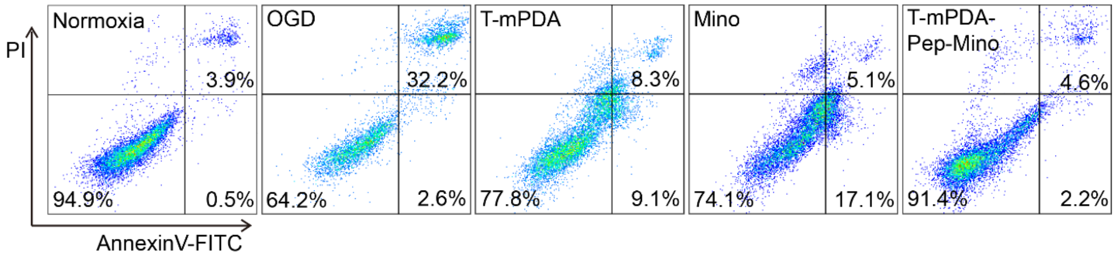

Supplementary Figure 11. Flow cytometry analysis of cellular apoptosis gating on AnnexinV-FITC and PI staining. Group normoxia was taken as the control test and the other four groups were treated by OGD, reperfusion and addition of saline, T-mPDA, Mino, and T-mPDA-Pep-Mino.

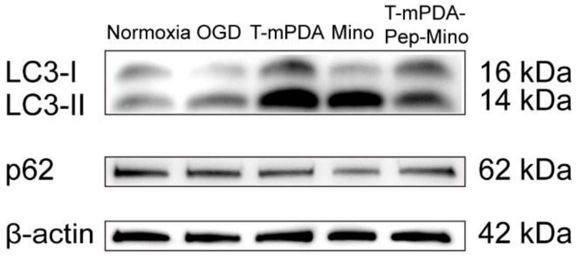

Supplementary Figure 12. Expression of autophagy-associated proteins by western blotting in different groups. More than 3 times are repeated independently with similar blots. Source data are provided as a Source Data file.

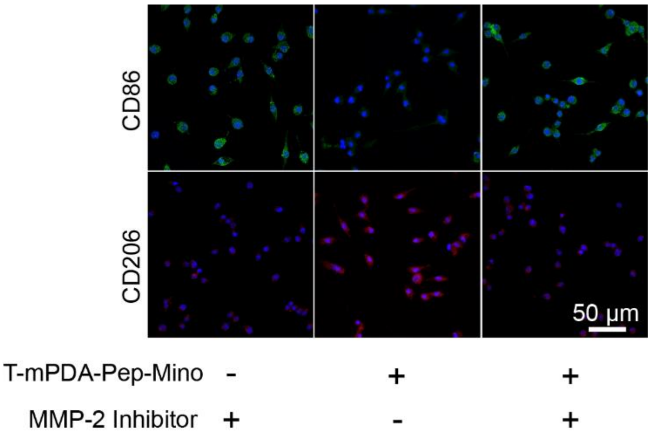

Supplementary Figure 13. Immunofluorescence staining of CD86 and CD206 of BV-2 cells incubated with or without the addition of T-mPDA-Pep-Mino and MMP-2 Inhibitor.

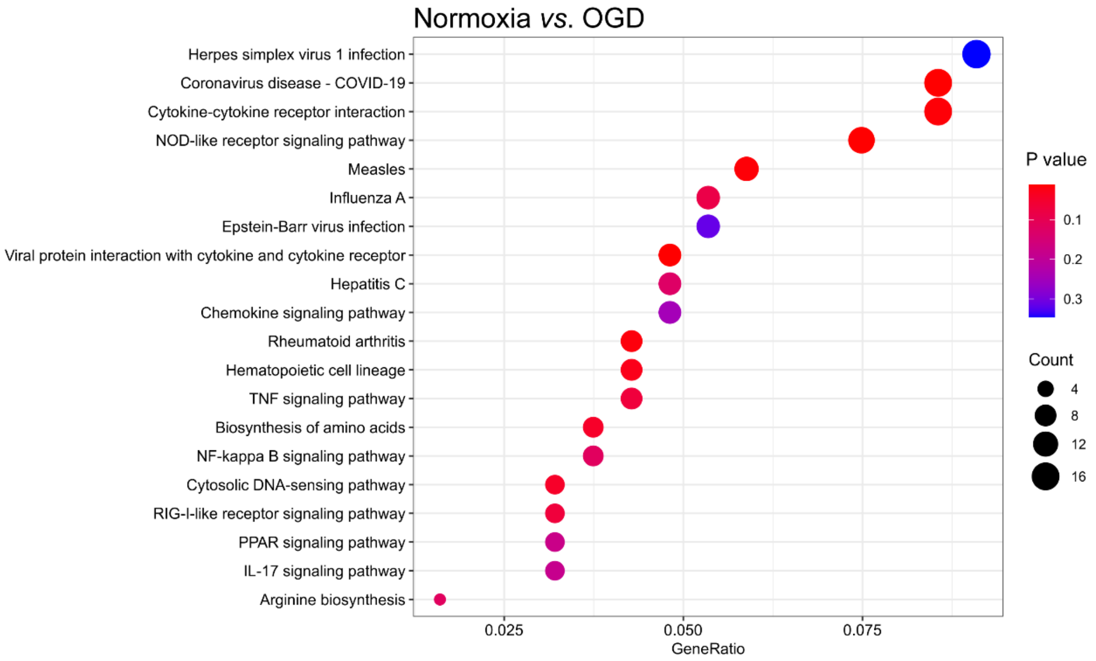

Supplementary Figure 14. Top 20 pathway enrichment determined through KEGG analysis between the groups of Normoxia and OGD.

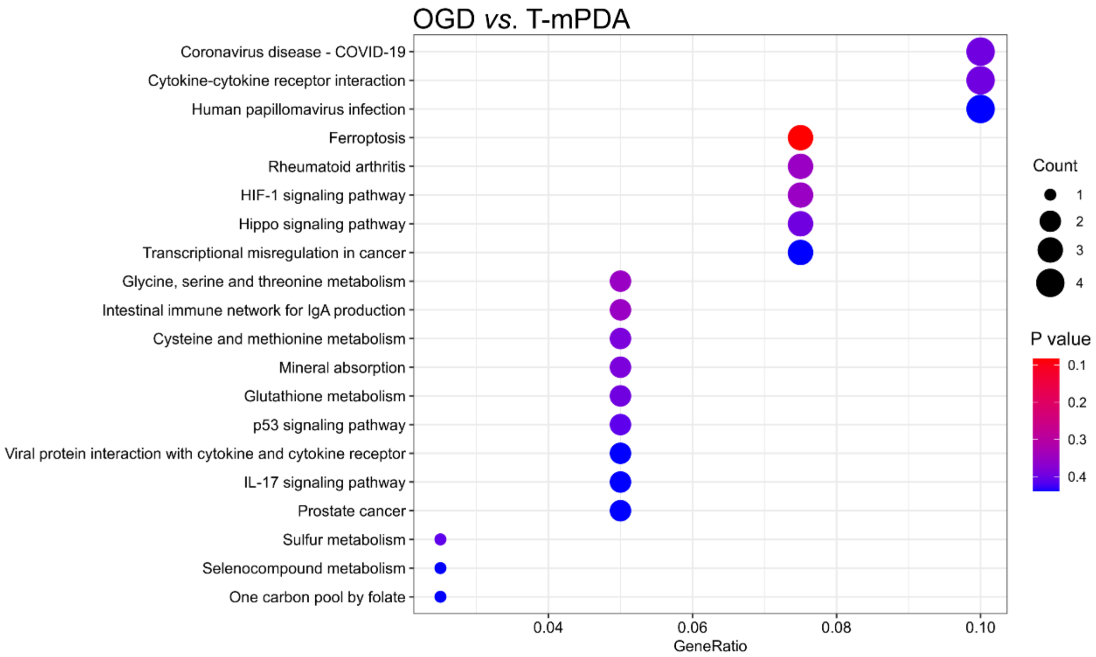

Supplementary Figure 15. Top 20 pathway enrichment determined through KEGG analysis between the groups of OGD and T-mPDA.

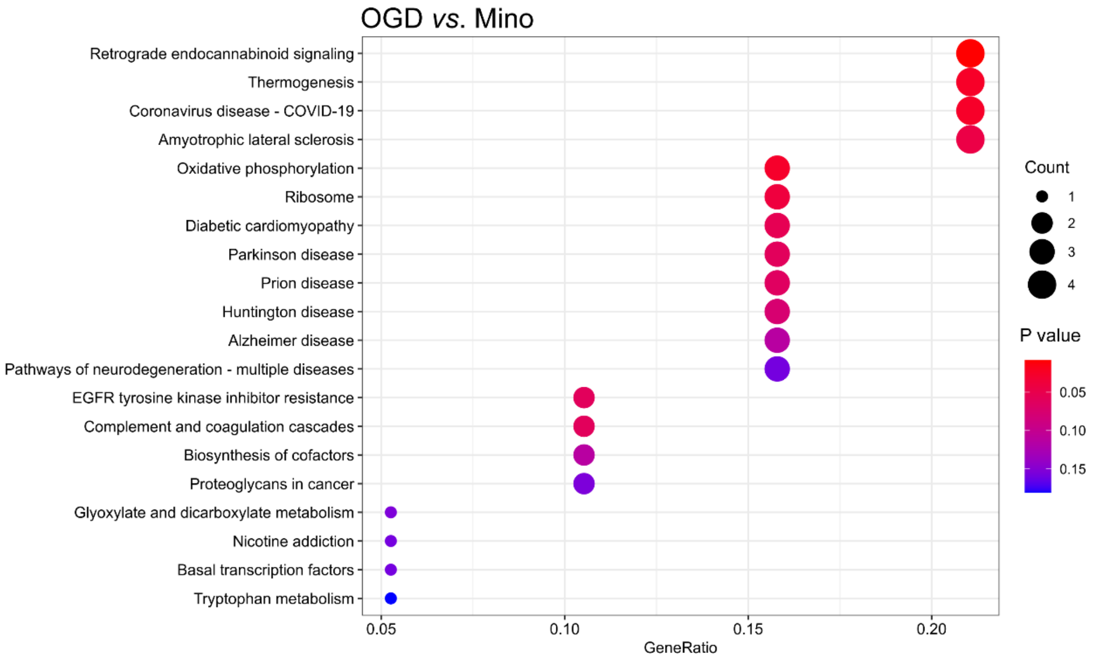

Supplementary Figure 16. Top 20 pathway enrichment determined through KEGG analysis between the groups of OGD and Mino.

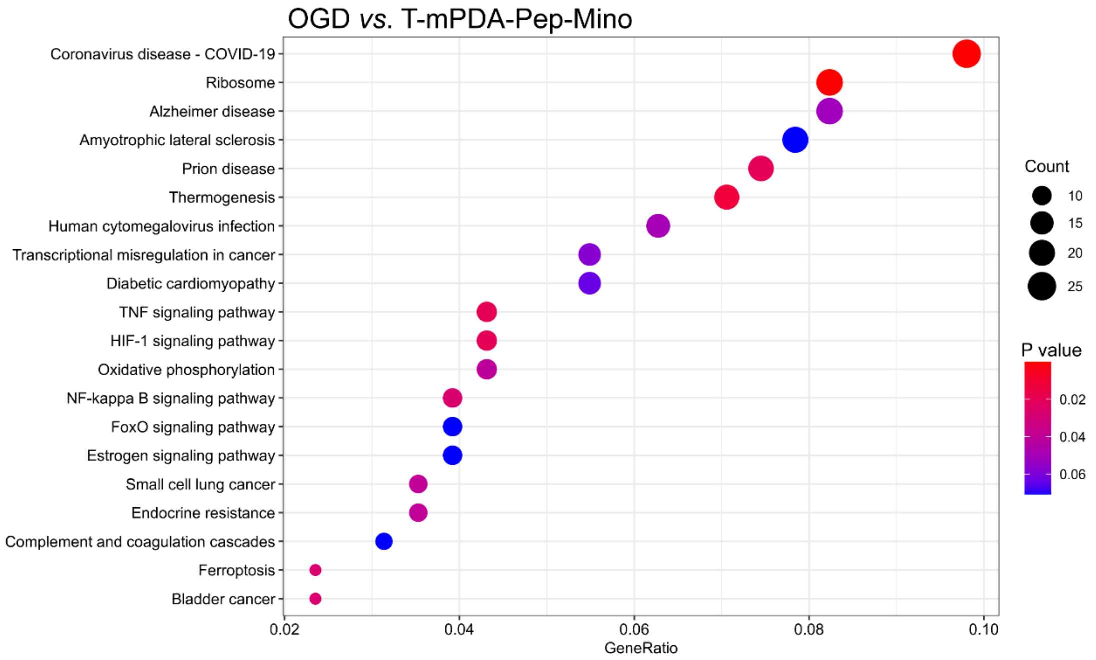

Supplementary Figure 17. Top 20 pathway enrichment determined through KEGG analysis between the groups of OGD and T-mPDA-Pep-Mino.

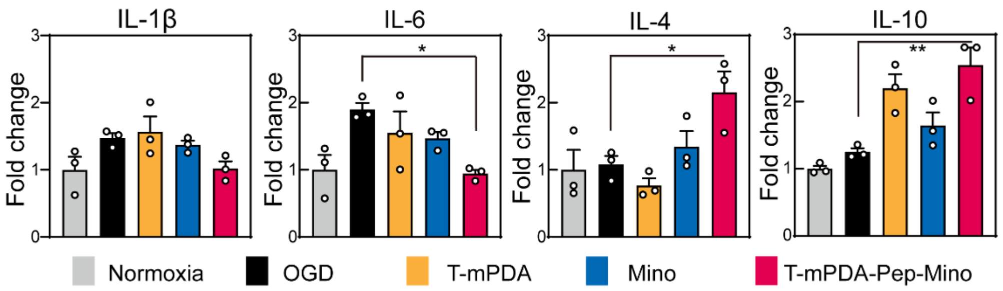

Supplementary Figure 18. Relative mRNA expression of IL-1β, IL-6, IL-4 and IL-10 in brain tissue from the mice in different groups determined by quantitative real-time PCR (n = 3 independent samples). (P* = 0.0287, P* = 0.0485, P** = 0.003 compared with the OGD group as indicated in the figures). The data are presented as means ± SEM. Source data are provided as a Source Data file.

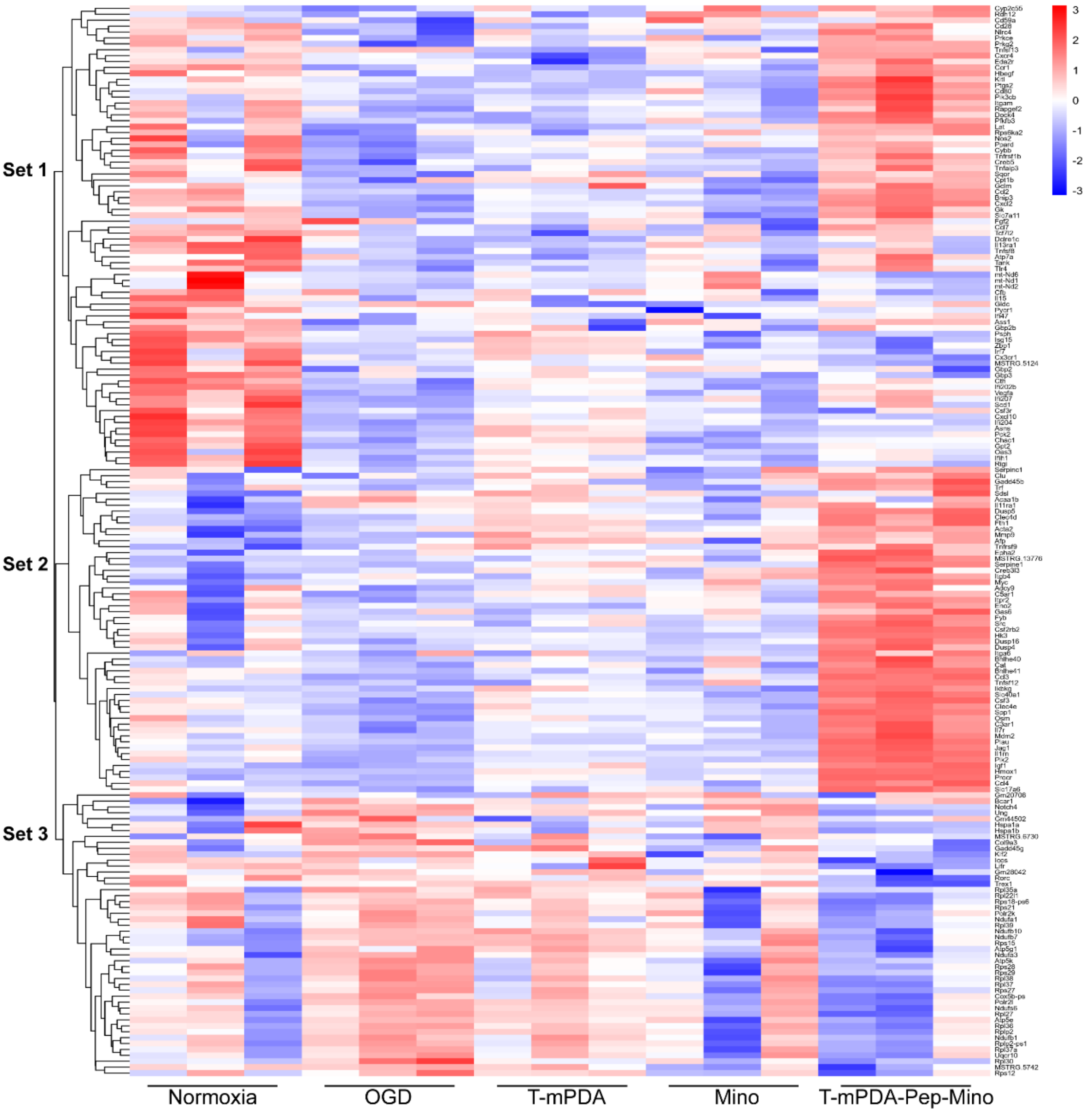

Supplementary Figure 19. An expression heatmap of genes in BV-2 cells under different treatments.

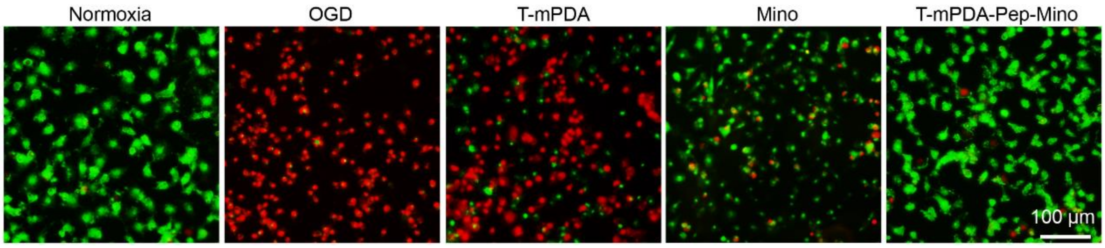

Supplementary Figure 20. Live/Dead fluorescence staining of neuron cells (incubated at the down chamber) in transwell study in different groups. Green and red fluorescence indicated the live and dead cells, respectively.

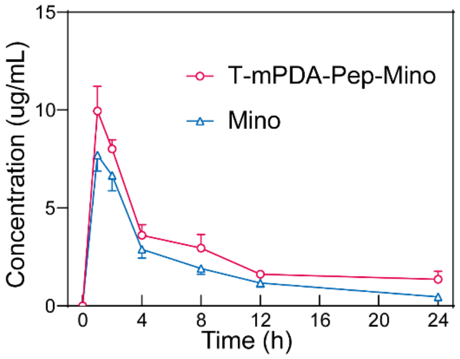

Supplementary Figure 21. Blood retention of T-mPDA-Pep-Mino and Mino at a dose of 10 mg/kg within 24 h after the drug administration (n = 3 independent samples). The data are presented as means ± SEM. Source data are provided as a Source Data file.

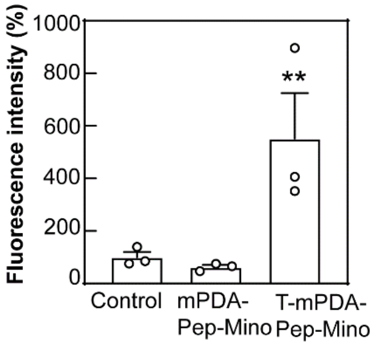

Supplementary Figure 22. Quantitative analysis of fluorescence intensity of ex vivo images of mice brains in different groups (n = 3 in independent samples). (P** = 0.0036 compared between the groups via Kruskal-Wallis test). The data are presented as means ± SEM. Source data are provided as a Source Data file.

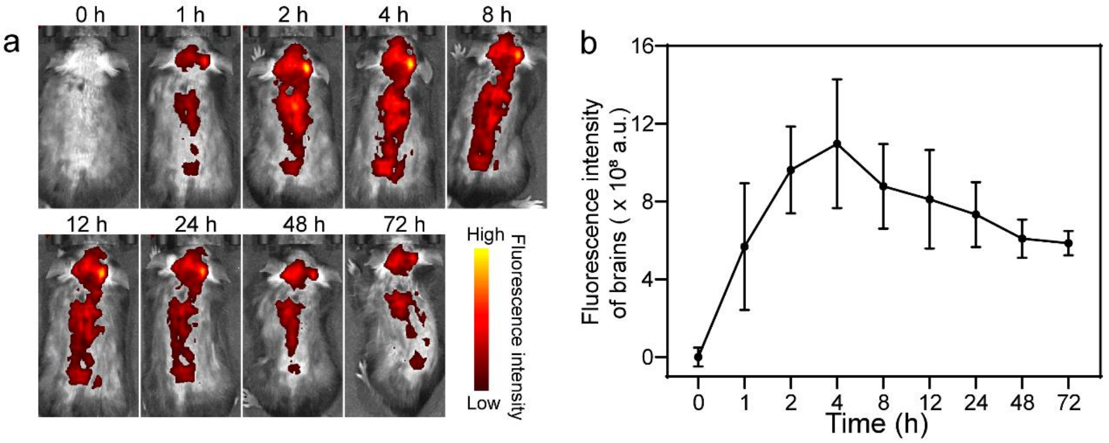

Supplementary Figure 23. In vivo imaging of MCAO mice treated with labelled T-mPDA-Pep-Mino and corresponding intensity analysis of the brain regions (n = 3 independent animals). The data are presented as means ± SEM. Source data are provided as a Source Data file.

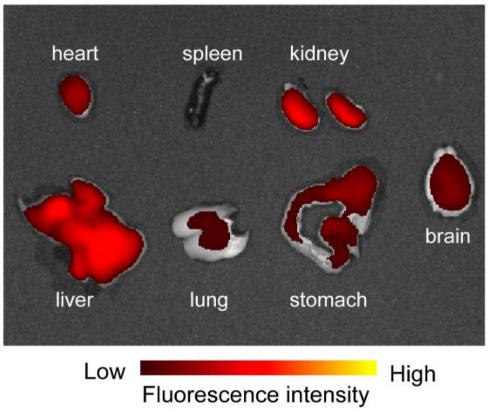

Supplementary Figure 24. Ex vivo imaging of the major organs of the MCAO mice treated with labelled T-mPDA-Pep-Mino.

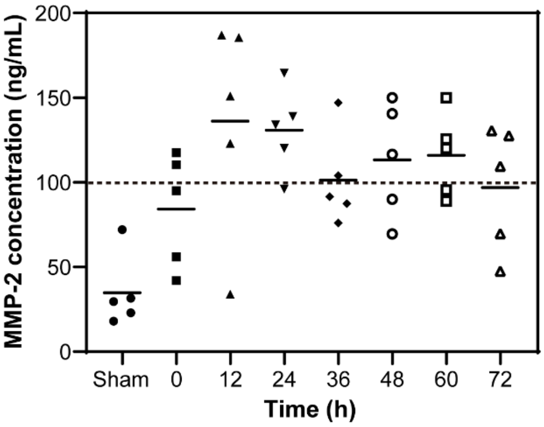

Supplementary Figure 25. MMP-2 concentration changes of the MCAO mice during the course of the ischemic event within three days post-reperfusion. The brain sections of euthanized MCAO mice were collected and the MMP-2 level was confirmed by ELISA assay (n = 5 independent

sample). Source data are provided as a Source Data file.

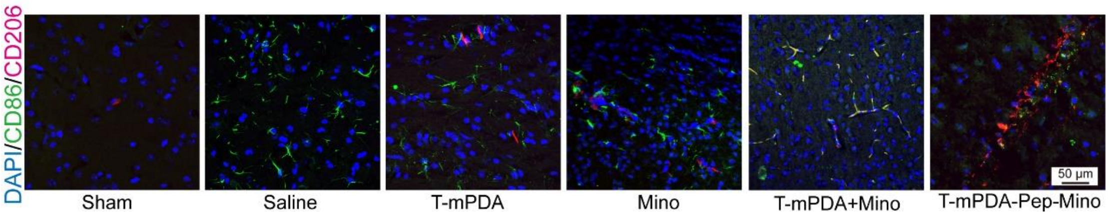

Supplementary Figure 26. Immunostaining of CD86 and CD206 of brain slices collected from the MCAO mice after different treatments.

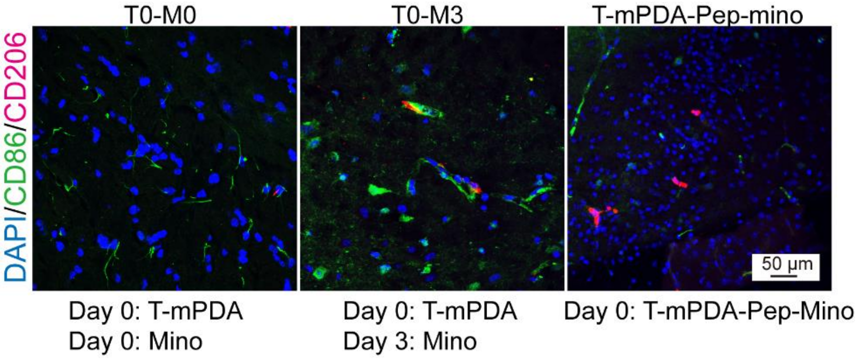

Supplementary Figure 27. Immunofluorescence staining of CD86 (green) and CD206 (red) of brain sections from the mice with different treatments. T0-M0: T-mPDA at day 0 and Mino at day 3; T0-M3: T-mPDA at day 0 and Mino at day. Cell nuclei were stained by DAPI.

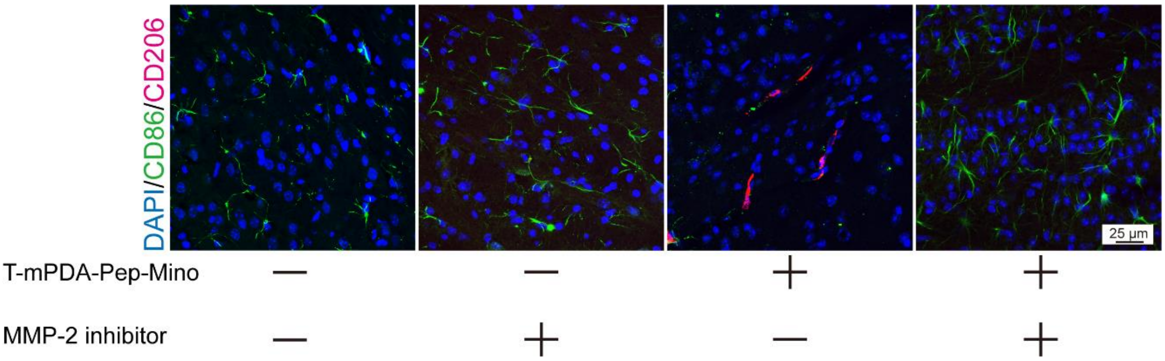

Supplementary Figure 28. Immunofluorescence staining of CD86 (green) and CD206 (red) of brain sections from the mice treated with or without T-mPDA-Pep-Mino or MMP-2 inhibitor. Cell nuclei were stained by DAPI.

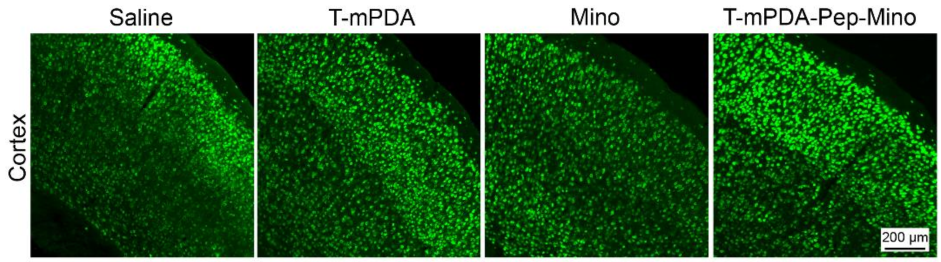

Supplementary Figure 29. Representative immunofluorescent images of neurons in cortex of brain slices in four groups. Neurons were stained with NeuN (green).

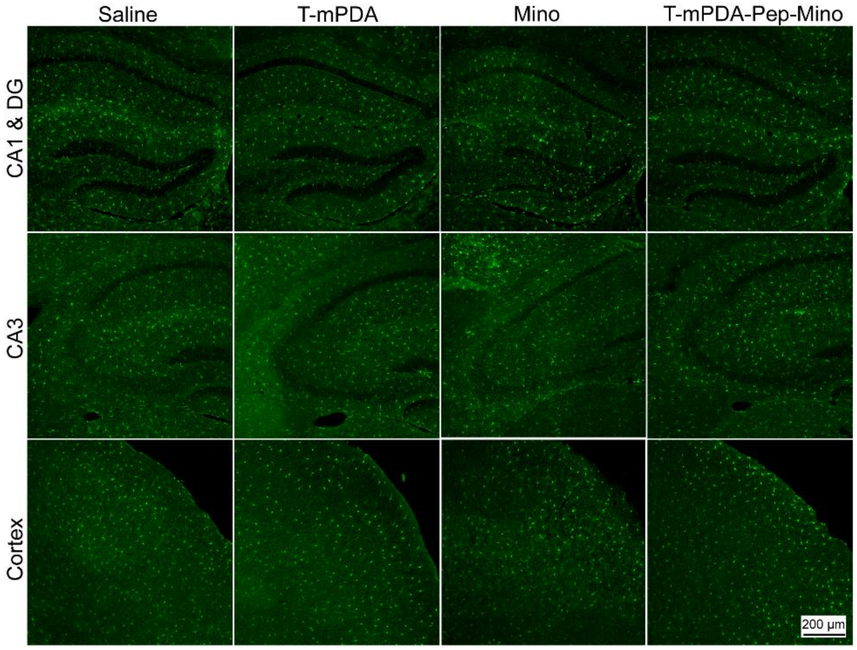

Supplementary Figure 30. Representative immunofluorescent images of microglia in CA1, CA3, DG, and cortex regions of brain slices in four groups. Microglia were stained with Iba-1 (green).

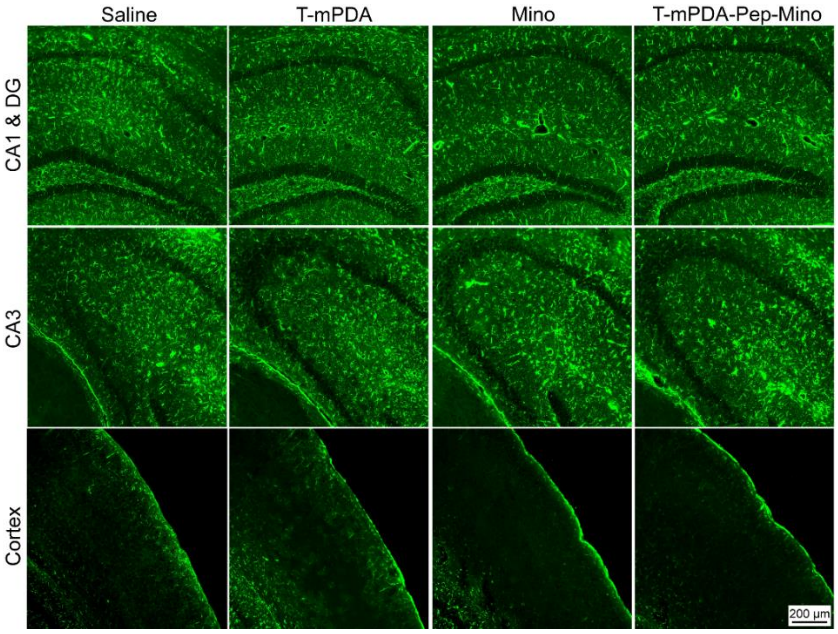

Supplementary Figure 31. Representative immunofluorescent images of astrocytes in CA1, CA3, DG, and cortex regions of brain slices in four groups. Astrocytes were stained with GFAP (green).

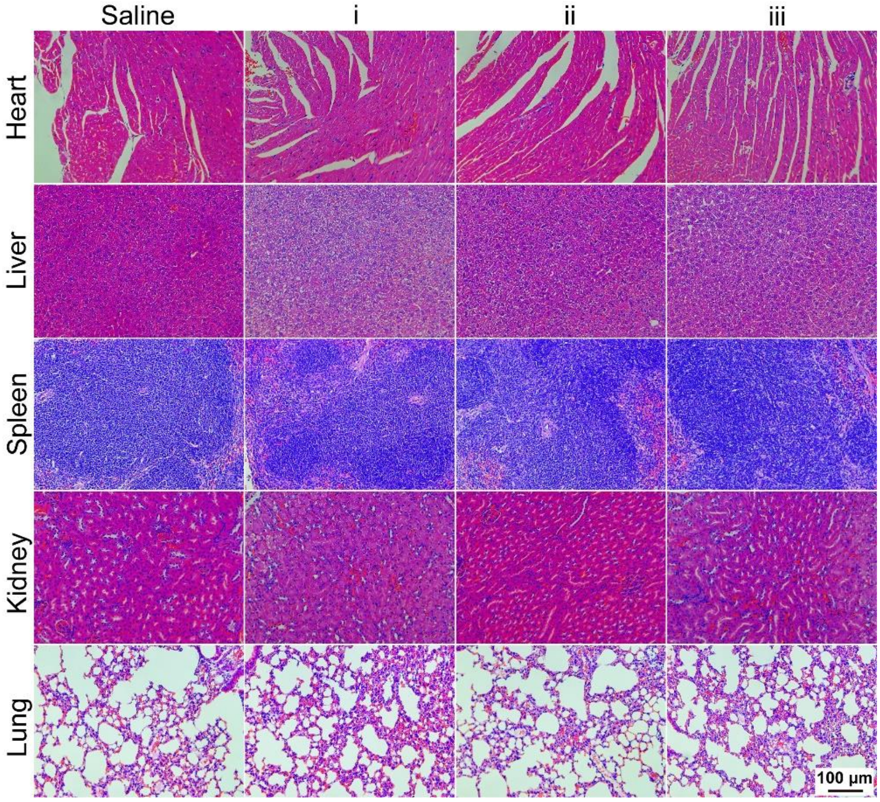

Supplementary Figure 32. Representative immunohistological images of major organs including heart, liver, spleen, lung, and kidney from the mice in different groups. Group saline, i, ii, and iii represent the groups of control, T-mPDA, Mino, and T-mPDA-Pep-Mino, respectively.

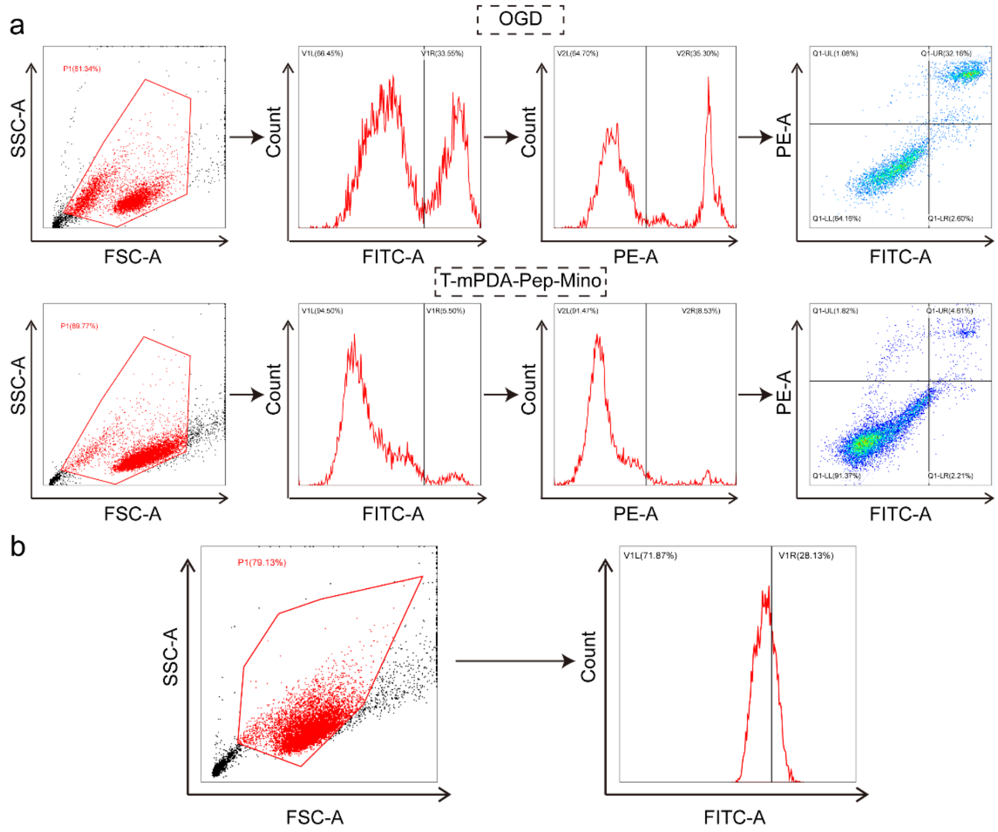

Supplementary Figure 33. Gating strategy of flow cytometry. The gating strategy of flow cytometry analysis of **a** cell apoptosis and **b** intracellular ionized Ca^{2+} levels in SH-SY5Y cells.

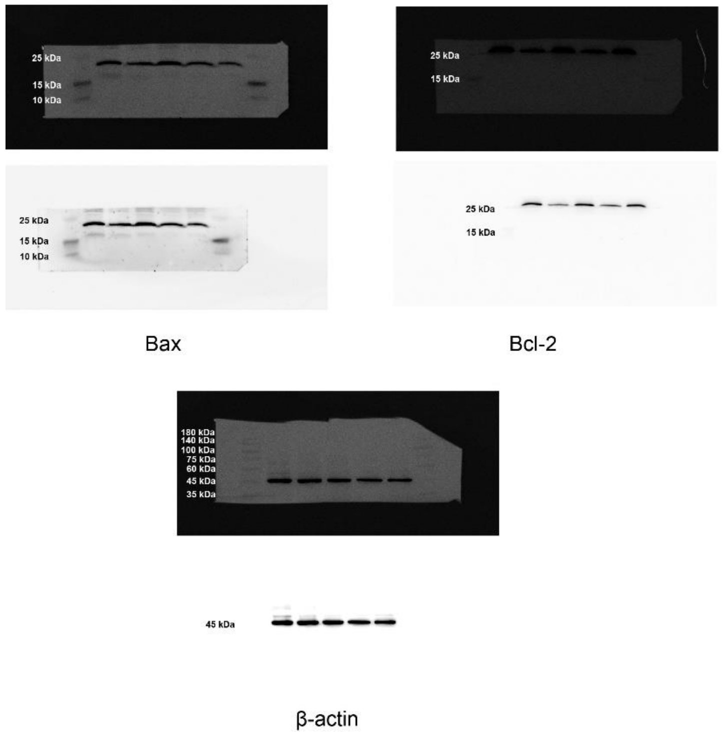

Supplementary Figure 34. Full uncropped scans of western blots images in Figure 2C for Bax, Bcl-2, and β-actin. Upper blots are obtained under white light and enhanced chemiluminescence

imaging; Bottom blots are captured under enhanced chemiluminescence imaging.

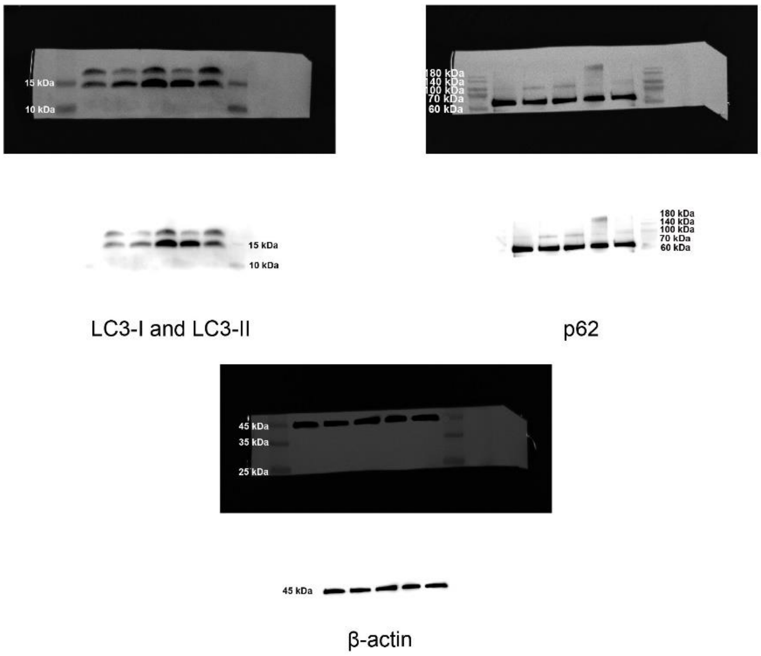

Supplementary Figure 35. Full uncropped scans of western blots images in Supplementary Figure 12 for LC3-I, LC3-II, p62, and β-actin. Upper blots are obtained under white light and enhanced chemiluminescence imaging; Bottom blots are captured under enhanced chemiluminescence imaging.
