# Supplementary material for: Pathogenesis-adaptive polydopamine nanosystem for sequential therapy of ischemic stroke
Source: Nat Commun. 2023 Nov 6;14:7147. doi: 10.1038/s41467-023-43070-z (PMC10628287; doi:10.1038/s41467-023-43070-z)
Supplement: Supplementary file 3 — Reporting Summary [file 41467_2023_43070_MOESM3_ESM.pdf]

nature portfolio

Corresponding author(s): Hongzhong Chen, Yanli Zhao, Xiaowei Zeng

Last updated by author(s): Oct 8, 2023

Reporting Summary

Nature Portfolio wishes to improve the reproducibility of the work that we publish. This form provides structure for consistency and transparency in reporting. For further information on Nature Portfolio policies, see our Editorial Policies and the Editorial Policy Checklist.

Statistics

For all statistical analyses, confirm that the following items are present in the figure legend, table legend, main text, or Methods section.

n/a	Confirmed	
☐	☒	The exact sample size (*n*) for each experimental group/condition, given as a discrete number and unit of measurement
☐	☒	A statement on whether measurements were taken from distinct samples or whether the same sample was measured repeatedly
☐	☒	The statistical test(s) used AND whether they are one- or two-sided<br>*Only common tests should be described solely by name; describe more complex techniques in the Methods section.*
☒	☐	A description of all covariates tested
☐	☒	A description of any assumptions or corrections, such as tests of normality and adjustment for multiple comparisons
☐	☒	A full description of the statistical parameters including central tendency (e.g. means) or other basic estimates (e.g. regression coefficient) AND variation (e.g. standard deviation) or associated estimates of uncertainty (e.g. confidence intervals)
☐	☒	For null hypothesis testing, the test statistic (e.g. *F*, *t*, *r*) with confidence intervals, effect sizes, degrees of freedom and *P* value noted<br>*Give P values as exact values whenever suitable.*
☒	☐	For Bayesian analysis, information on the choice of priors and Markov chain Monte Carlo settings
☒	☐	For hierarchical and complex designs, identification of the appropriate level for tests and full reporting of outcomes
☒	☐	Estimates of effect sizes (e.g. Cohen's *d*, Pearson's *r*), indicating how they were calculated

Our web collection on statistics for biologists contains articles on many of the points above.

Software and code

Policy information about availability of computer code

Data collection	The hydrodynamic diameter, zeta potential, and polydispersity index (PDI) were measured by dynamic light scattering (DLS) using a Malvern Mastersizer 2000 instrument;<br>TEM and SEM images were captured by FEI Tecnai G2 F30 TEM and Regulus 8230 SEM, respectively;<br>Cell and tissue immunofluorescent staining images and Live/Dead observation of biofilm were collected from Zeiss LSM 880 confocal microscope with built-in software ZEN 2.0 (blue edition) and ZEN (black edition);<br>Flow cytometry data were collected by Beckman cytoflex with flowJo (Vision 10.6.2) for data collection;<br>The data related to fluorescence intensity and absorbance were measured using an enzyme-linked immunosorbent assay (ELISA) analyzer (Tecan/Infinite E plex);<br>EPR data was collected by Bruker EMXplus;<br>q-PCR was collected by ABI QuantStudio 5;<br>The selenium content was detected using PerkinElmer Optima 8300.
Data analysis	Graphpad Prism 9.0.0 and Origin 2022b were used to analysed data.<br>Cell and tissue immunofluorescence staining images were analyzed with ZEN 2.0 (blue edition).<br>Grayscale intensity of the images and wound data were quantified using the Image J 1.53.<br>Biomass and thickness of biofilms were quantified using the Comstat 2.1.

For manuscripts utilizing custom algorithms or software that are central to the research but not yet described in published literature, software must be made available to editors and reviewers. We strongly encourage code deposition in a community repository (e.g. GitHub). See the Nature Portfolio guidelines for submitting code & software for further information.

Data

Policy information about availability of data

All manuscripts must include a data availability statement. This statement should provide the following information, where applicable:

- Accession codes, unique identifiers, or web links for publicly available datasets
- A description of any restrictions on data availability
- For clinical datasets or third party data, please ensure that the statement adheres to our policy

Source data are provided with this paper. All other data are available from the corresponding authors upon request.

Human research participants

Policy information about studies involving human research participants and Sex and Gender in Research.

Reporting on sex and gender	N/A
Population characteristics	N/A
Recruitment	N/A
Ethics oversight	N/A

Note that full information on the approval of the study protocol must also be provided in the manuscript.

Field-specific reporting

Please select the one below that is the best fit for your research. If you are not sure, read the appropriate sections before making your selection.

☒ Life sciences ☐ Behavioural & social sciences ☐ Ecological, evolutionary & environmental sciences

For a reference copy of the document with all sections, see nature.com/documents/nr-reporting-summary-flat.pdf

Life sciences study design

All studies must disclose on these points even when the disclosure is negative.

Sample size	Sample size were determined according to the pilot and on the basis of the previous experimental experiences (Nature communications, 2022, 13, 3875; Science Advances, 2020, 6: eabb0025). Generally three independent replicates were done for in vitro experiments, and four to six independent biological replicates for in vivo experiments.
Data exclusions	No data was excluded from any of the analyses in this work.
Replication	All in vitro experiments were repeated independently for at least 3 times. All in vivo experiments were repeated with at least 3 mice: 6 mice per group for analyzing the wound healing and 3 mice per group for histological analysis and immune cell analysis. This information is also given in the figure captions and Methods section.
Randomization	For all studies, samples were randomly divided into different experimental groups.
Blinding	No formal blinding was used in this study. Because all experiments were performed based on standardized protocols and blinding has no effect on the experiment results. Unbiased experimental procedure and data analysis were carried out as far as possible.

Reporting for specific materials, systems and methods

We require information from authors about some types of materials, experimental systems and methods used in many studies. Here, indicate whether each material, system or method listed is relevant to your study. If you are not sure if a list item applies to your research, read the appropriate section before selecting a response.

Materials & experimental systems

n/a	Involved in the study
☐	☒ Antibodies
☐	☒ Eukaryotic cell lines
☒	☐ Palaeontology and archaeology
☐	☒ Animals and other organisms
☒	☐ Clinical data
☒	☐ Dual use research of concern

Methods

n/a	Involved in the study
☒	☐ ChIP-seq
☐	☒ Flow cytometry
☒	☐ MRI-based neuroimaging

Antibodies

Antibodies used

APC anti-mouse CD197, Biolegend, 120107, FC, 1:200
FITC anti-mouse CD206, Biolegend, 141703, FC, 1:200
PE anti-mouse F4/80, Biolegend, 123109, FC, 1:200
GPX4 Rabbit pAb, ABclonal, A13309, WB, 1:500
STAT6 Antibody, Affinity, AF6302, WB, 1:500
Phospho-STAT6 (Tyr641) Antibody, Affinity, AF3301, WB, 1:500
JAK1 Antibody, Affinity, AF5012, WB, 1:500
Phospho-JAK1 (Tyr1022/Tyr1023)[Tyr1034/Tyr1035] Antibody, Affinity, AF2012, WB, 1:500
ERK1/2 Antibody, Affinity, AF0833, WB, 1:500
Phospho-ERK1/2 (Thr202/Tyr204) Antibody, Affinity, AF1015, WB, 1:500
CD86 (E5W6H) Rabbit mAb, Cell signaling technology, 19589, IF, 1:200
Goat anti-rabbit IgG (HRP), Abcam, ab205718, IF, 1:4000

Validation

APC anti-mouse CD197 has been validated to be used for flow cytometric analysis and mentioned species reactivity with mouse (https://www.biolegend.com/en-us/products/apc-anti-mouse-cd197-ccr7-antibody-2822)
FITC anti-mouse CD206 has been validated to be used for flow cytometric analysis and mentioned species reactivity with mouse (https://www.biolegend.com/en-us/products/fitc-anti-mouse-cd206-mmr-antibody-7318)
PE anti-mouse F4/80 has been validated to be used for flow cytometric analysis and mentioned species reactivity with mouse (https://www.biolegend.com/en-us/products/pe-anti-mouse-f4-80-antibody-4068)
STAT6 Antibody has been validated to be used for Western blot analysis and mentioned species reactivity with mouse (https://www.affbiotech.cn/goods-1899-AF6302-STAT6_Antibody.html)
Phospho-STAT6 (Tyr641) Antibody has been validated to be used for Western blot analysis and mentioned species reactivity with mouse (https://www.affbiotech.cn/goods-1466-AF3301-Phospho_STAT6_Tyr641_Antibody.html)
JAK1 Antibody has been validated to be used for Western blot analysis and mentioned species reactivity with mouse (https://www.affbiotech.cn/goods-1679-AF5012-JAK1_Antibody.html)
Phospho-JAK1 (Tyr1022/Tyr1023)[Tyr1034/Tyr1035] Antibody has been validated to be used for Western blot analysis and mentioned species reactivity with mouse (https://www.affbiotech.cn/goods-1183-AF2012-Phospho_JAK1_Tyr1022_Tyr1023_Tyr1034_Tyr1035_Antibody.html)
ERK1/2 Antibody has been validated to be used for Western blot analysis and mentioned species reactivity with mouse (https://www.affbiotech.cn/goods-866-AF0833-ERK1_2_Antibody.html)
Phospho-ERK1/2 (Thr202/Tyr204) Antibody has been validated to be used for Western blot analysis and mentioned species reactivity with mouse (https://www.affbiotech.cn/goods-1178-AF1015-Phospho_ERK1_2_Thr202_Tyr204_Antibody.html)
CD86 (E5W6H) Rabbit mAb has been validated to be used for immunohistochemistry analysis and mentioned species reactivity with mouse (https://www.cellsignal.cn/products/primary-antibodies/cd86-e5w6h-rabbit-mab/19589?site-search-type=Products&N=4294956287&Ntt=19589&fromPage=plp&_requestid=14425),
Goat anti-rabbit IgG (HRP) has been validated to be used for immunohistochemistry analysis and mentioned species reactivity with mouse (https://www.abcam.cn/products/secondary-antibodies/goat-rabbit-igg-hl-hrp-ab205718.html)

Eukaryotic cell lines

Policy information about cell lines and Sex and Gender in Research

Cell line source(s)	RAW264.7 and HUVEC were obtained form American Type Culture Collection (ATCC)
Authentication	No authentication was performed.
Mycoplasma contamination	Cell lines were tested negative for mycoplasma contamination.
Commonly misidentified lines (See ICLAC register)	No cell lines used are listed in the database of commonly misidentified cell lines.

Animals and other research organisms

Policy information about studies involving animals; ARRIVE guidelines recommended for reporting animal research, and Sex and Gender in Research

Laboratory animals	The BALB/c (5-6 week old) male mice were purchased fromLaboratory Animal Center of Sun Yat-Sen University. All the mice (five mice per cage) were housed in standard, infection-free housing room, with 12h light:12h dark cycles in the vivarium at the Sun Yat-Sen University.
Wild animals	No wild animal was used in this study.
Reporting on sex	Male mice were used to model diabetic chronic wounds because the incidence of diabetes is higher in males than in females
Field-collected samples	This study did not involve samples collected from the field.
Ethics oversight	All procedures and animal cares were in accordance with the animal protocol approved by Institutional Animal Care and Use Committee of Sun Yat-Sen University (SYSU-IACUC-2022-000945)

Note that full information on the approval of the study protocol must also be provided in the manuscript.

Flow Cytometry

Plots

Confirm that:

- [x] The axis labels state the marker and fluorochrome used (e.g. CD4-FITC).
- [x] The axis scales are clearly visible. Include numbers along axes only for bottom left plot of group (a 'group' is an analysis of identical markers).
- [x] All plots are contour plots with outliers or pseudocolor plots.
- [x] A numerical value for number of cells or percentage (with statistics) is provided.

Methodology

Sample preparation	RAW264.7 cells from different groups were collected and stained for flow cytometry
Instrument	Beckman cytoflex
Software	FlowJo V10.6.2 was used for data analysis
Cell population abundance	The relative abundance was maintained by diluting all the samples at equal volume and collecting samples at a fixed and consistent time.
Gating strategy	All cells were selected except for the debris.

- [x] Tick this box to confirm that a figure exemplifying the gating strategy is provided in the Supplementary Information.
